# Repository of proposed pathways and protein–protein interaction networks in age-related macular degeneration

**DOI:** 10.1038/s41514-019-0039-5

**Published:** 2020-01-07

**Authors:** Fran M. Pool, Christina Kiel, Luis Serrano, Philip J. Luthert

**Affiliations:** 1 UCL Institute of Ophthalmology, and NIHR Moorfields Biomedical Research Centre, University College London, 11-43 Bath Street, London, EC1V 9EL UK; 2 Systems Biology Ireland & Charles Institute of Dermatology & School of Medicine, University College Dublin, Belfield Dublin, 4 Ireland; 3 Centre for Genomic Regulation (CRG), Systems Biology Programme. The Barcelona Institute of Science and Technology, Dr. Aiguader 88, Barcelona, 08003 Spain; 4 Universitat Pompeu Fabra (UPF), Barcelona, 08003 Spain; 5 Institució Catalana de Recerca i Estudis Avançats (ICREA), Pg. Lluis Companys 23, Barcelona, 08010 Spain

**Keywords:** Proteins, Ageing

## Abstract

Age-related macular degeneration (AMD) is one of the commonest causes of sight loss in the elderly population and to date there is no intervention that slows or prevents early AMD disease progressing to blinding neovascularization or geographic atrophy. AMD is a complex disease and factors proposed to contribute to the development and progression of disease include aging, genetics, epigenetics, oxidative stress, pro-inflammatory state, and life-style factors such as smoking, alcohol, and high fat diet. Here, we generate a knowledge repository of pathways and protein–protein interaction (PPI) networks likely to be implicated in AMD pathogenesis, such as complement activation, lipid trafficking and metabolism, vitamin A cycle, oxidative stress, proteostasis, bioenergetics, autophagy/mitophagy, extracellular matrix (ECM) turnover, and choroidal vascular dropout. Two disctinct clusters ermerged from the networks for parainflamation and ECM homeostasis, which may represent two different disease modules underlying AMD pathology. Our analyses also suggest that the disease manifests primarily in RPE/choroid and less in neural retina. The use of standardized syntax when generating maps of these biological processes (SBGN standard) and networks (PSI standard) enables visualization of complex information in graphical programs such as CellDesigner and Cytoscape and enhances reusability and extension of data. The ability to focus onto subnetworks, multiple visualizations and simulation options will enable the AMD research community to computationally model subnetworks or to test experimentally new hypotheses arising from connectivities in the AMD pathway map.

## Introduction

By the age of 65, it is thought that 1 in every 3 people will have some form of vision loss due to an eye disease.^1,2^ The most common of these diseases is age-related macular degeneration (AMD).^3^ There is currently no cure for AMD and there is no intervention that slows or prevents early AMD disease progressing to late AMD whether manifest as choroidal neovascularization or geographic atrophy. Patients with AMD generally become symptomatic when cells in the center of the retina degenerate or when new blood vessels form at the same location. Patients report distorted, smudged, or absent central vision and have problems with reading, driving, and recognizing faces. One of the first clinical characteristics of AMD is a functional reduction in rod photoreceptor recovery time following bright bleaching stimulus—manifest as loss of dark adaptation, pointing toward an interruption in the visual cycle. Structurally, the only early abnormality in AMD may be the presence of small drusen, between the retinal pigment epithelium (RPE) and Bruch’s membrane. These are associated with a relatively low risk of disease progression. Intermediate AMD is characterized by one or more large drusen at the macula and/or pigmentary change that has no other explanation and is associated with an increased risk of progression to visual impairment,^4^ which can arise in two main ways. If blood vessels migrate from the choroid (or more rarely the retina) into the sub-RPE and/or subretinal space the fibrovascular tissue and associated fluid leakage can distort vision and if hemorrhage occurs, visual loss can be sudden. In so-called geographic atrophy there is concurrent loss of photoreceptors, RPE and choriocapillaris, leading to central vision loss. Reticular pseudodrusen (sometimes called subretinal drusenoid deposits, but these may not be precisely the same thing) are a relatively recently defined phenotype^5^ that is seen in AMD as well as other outer retinal degenerations characterized by Bruch’s membrane/RPE pathology.

AMD is clearly a multifactorial, multi-compartmental complex disease.^6^ While a rich and complex analysis of the genetic contribution to the condition has been elucidated,^7^ the penetrance of many of these variants is very low, and one of the challenges of complex disease is to capture the breadth of processes that may be perturbed. This challenge is made greater when considering complex disease such as AMD (or Alzheimer’s disease, other neurodegenerative conditions and cardiovascular disease) because they exist in the context of aging. Genetic studies have shown that complement activation is likely to be a factor in the pathogenesis of AMD and there is also high risk associated with polymorphisms at the rather enigmatic ARMS2/HTRA1 locus on chromosome 10. Smoking is a risk factor and individuals without genetic or clearly defined environmental risk can get disease, reinforcing the view that AMD is a complex age-related disorder. Some of the pathways implicated in its pathogenesis are complement activation,^8^ lipid trafficking and metabolism,^9,10^ vitamin A cycle,^11,12^ oxidative stress,^13,14^ proteostasis,^15^ bioenergetics,^16^ autophagy/mitophagy,^17^ ECM turnover,^18^ endothelial cell dropout in the choriocapillaris.^19^

Network-centric approaches in medicine have been used extensively to capture the molecular complexity of human disease by representing interconnectivities of cellular components, such as proteins, metabolites, and RNA in the form of networks, biochemical reactions, and pathways.^20^ A detailed understanding of networks and pathways perturbed in a disease is critical to develop better targets for drug development. However, no single network methodology provides a comprehensive description. Therefore, here we used two different network-centric approaches to capture the potential molecular mechanisms of AMD pathogenesis. A representation in SBGN (systems biology graphical notation)^21^ was used to generate an anatomically informed interaction and biochemical reaction map. This visual representation—created using the software CellDesigner^22^—enables visualization of disease pathways, their associations and their distribution across different anatomical compartments. We considered the precise spatial organization of cellular and extracellular components of the posterior pole of the eye in recognition of the importance of the exquisite anatomy of the choroid, Bruch’s membrane, RPE, and photoreceptors. The other, complementary network was centered around a set of 110 AMD-risk gene products (proteins) expressed in retinal and choroidal cell types, which link to 1426 proteins via direct binary protein interactions that were retrieved from databases and literature searches. This protein–protein interaction (PPI) network—represented in Cytoscape^23^—highlights associations to homeostasis functions, such as proteostasis, energy homeostasis/mitophagy, ECM homeostasis, autophagy, cholesterol homeostasis, redox balance and control of reactive oxygen species, phagocytosis, choroidal vascular homeostasis, and lipid homeostasis. We propose that the combination of disease representations will enable greater insights into disease pathogenesis. The AMD maps are publicly available in a UCL research data repository (see Data Availability for details).

## Results

### Representation of AMD in SBGN

**Fig. 1:**
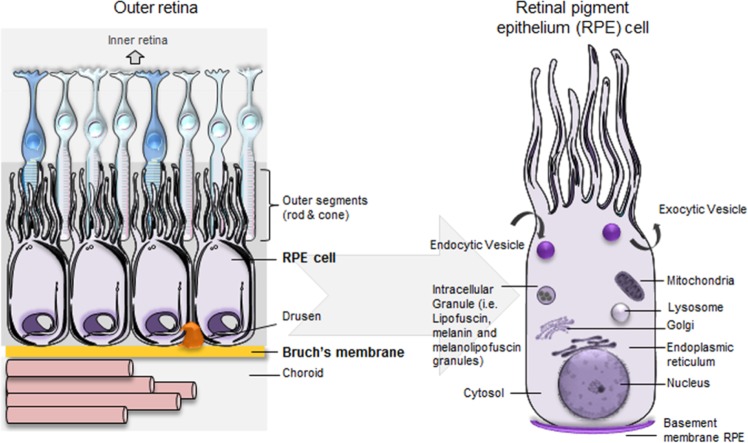
Compartments created in the CellDesigner platform in line with the structure and function of the outer retina. The outer retina lies internal to a layer of fenestrated choroidal capillaries called the choriocapillaris that supply the retina with oxygen, lipoproteins, glucose, vitamins and other nutrients, as well as removing waste substances such as carbon dioxide and lactate. The connective tissue layer between the choriocapillaris and the RPE is the Bruch’s Membrane. It is made up of five layers containing ECM proteins and acts rather like a sieve, providing a degree of constraint to the reciprocal exchange of molecules, nutrients, fluids, and waste products between the retina and circulation. The RPE is made up of a single layer of hexagonal cells and serves many functions. With tight junctions between the lateral surfaces, the RPE functions as part of the blood-ocular barrier, isolating the inner eye from systemic influences. Separating the inner eye from circulation provides immune privilege protecting the vital cells and structures from potential damage or loss of function via inflammatory immune response. The RPE is responsible for the selective transport and processing of all nutrients, metabolites, signaling molecules, and waste products to and from the inner retina, including the photoreceptors. Note that compartments are not drawn to scale.

**Fig. 2:**
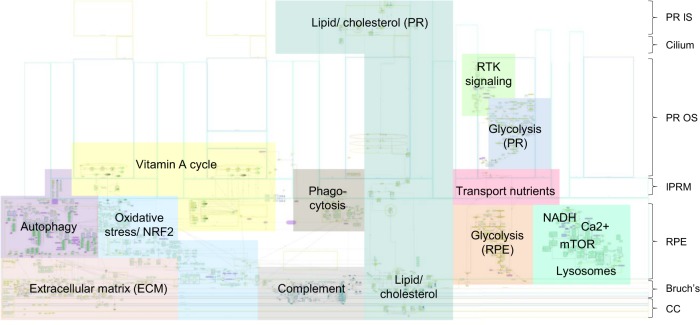
Illustration of the full SBGN model of AMD pathogenesis. The pathway map was generated using CellDesigner.^22^ For additional map details see Data Availability, Supplementary File 1 (can be viewed using CellDesigner software).

The first step in building the model was to define the spatial compartments to be explicitly represented. We defined multiple spatial compartments in line with the structure and function of the outer retina and choroid (Fig. 1, Table 1).^24^ We also included compartments relevant to AMD pathogenesis including sub-RPE deposits such as drusen and macrophages which are seen as part of the inflammatory landscape of AMD.^25^ Next, to assign processes implicated in AMD pathogenesis to each compartment and to build the SBGN model, several strategies to select information from the literature were adopted. Acknowledging that there is no good animal model for AMD, we concentrated on human data arising from cell culture, genetics, biochemistry, experimental medicine, and histopathological studies. Reviews of the AMD pathogenesis were surveyed for pathways suggested to be involved as well as specific experimental papers with an emphasis on human data. As certain fundamental physiological mechanisms such as dark adaptation/vitamin A cycling between RPE and photoreceptors are disrupted in AMD, these were also included. We also considered risk pathways identified from a recent GWAS study,^7^ as well as our own assessment of potentially informative risk genes (see Methods). The resulting constructed SBGN model integrates elements of gene regulation, signaling processes and metabolic reactions leading to a large and comprehensive network interaction model. To keep this model consistent with pre-existing databases we used official gene names throughout. In all, we have collected and curated 111 reviews/publications/websites detailing AMD and its pathogenesis (Supplementary Data 1). The final AMD SBGN model contains 1098 species (164 complexes, 575 proteins, 103 RNA, 130 simple molecules, 62 phenotypes, 10 ions, 1 drugs, 2 genes, and 51 degradations) and 1022 reactions (Fig. 2; see Data Availability, Supplementary File 1).

**Table 1:** Compartments created in the CellDesigner platform in line with the structure and function of the outer retina.

Outer retina anatomical compartments	Substructures within anatomical compartments	Further subclassification of anatomical structures and organelles	Organelles and deposits in anatomical substructures
Choroid	Choriocapillaris	Lumen	Macrophage
	Choriocapillaris	Endothelium	Mitochondria
	Bruch’s membrane	Outer collagen free zone	
		Outer collagenous layer	
		Elastin layer	
		Inner collagenous layer	
		Inner collagen free zone	Hard drusen
			Soft drusen/basal linear deposit
RPE	RPE basement membrane	Basal laminar deposit	
		Exosome	
	RPE	Nucleus	
		Endoplasmic reticulum	
		Golgi	
		Mitochondria	
		Endocytic vesicle	
		Transcytotic vesicle	
		Transport vesicle	
		Early endosome	
		Recycling endosome	
		Late endosome	
		Lysosome	
		Phagosome	
		Phagolysosome	
		Autophagosome	
		Melanosome	
		Secretory vesicle	
Interphotoreceptor Matrix	Reticular subretinal drusenoid deposit		
	Macrophage		
Photoreceptor	Rod outer segment	Disks	
	Shed rod outer segment	Disks	
	Cilium		
	Rod inner segment	Endocytic vesicle	
		Mitochondria	
	Endoplasmic reticulum		
	Nucleus

Mitochondrial DNA is particularly susceptible to oxidative damage in human RPE.^26^ Combined with the reduction of cellular antioxidant system capacity due to aging^27^ it seems likely that mitochondria will have an important role in the pathogenesis of AMD and so we considered it important to include mitochondria in the graphical representation (see Data Availability, Supplementary File 2). The accumulation of macrophages is described as a feature of early and late AMD,^25^ and although the role of macrophages in pathogenesis is not fully defined, we considered it important to include a subretinal macrophage submodule (see Data Availability, Supplementary File 3). These graphical models were adapted from published models linked to mapping of Parkinson’s disease pathogenesis after filtering according to gene-expression data in the retina and RPE.^28^

### The AMD pathogenesis in SBGN and subnetworks

The SBGN map (see Data Availability, Supplementary File 1) is oriented with photoreceptors at the top and the choriocapillaris at the base (overview in Fig. 2). At the left-hand end of Bruch’s membrane is a summary list of molecules found at this site.^29^ To the right of this is a dense network capturing the complex interactions between different extracellular matrix (ECM) components and associated proteases and protease inhibitors as well as some of the trafficking between Bruch’s membrane and the overlying RPE. The latter includes recycling with the early endosome compartment, the liberation of matrix metalloproteinases from intracellular granules. Key subnetworks of the SBGN representation of the complexity of AMD pathogenesis are autophagy, vitamin A cycle, transport of nutrients, complement activation, ECM turnover, lipid/cholesterol trafficking and metabolism, and energy metabolism (mitochondria). Some of the above-mentioned subnetworks overlap. For example, some nutrients, complement proteins, vitamin A precursors and lipids/cholesterol are transported in lipoprotein particles as well as in the form of discrete molecules. Furthermore, complement activation may play a role in ECM turnover, which in turn may play a role in the transport of lipoproteins across Bruch’s membrane.

### PPI network centered around risk proteins

As a complementary approach to capture potential molecular mechanisms of AMD pathogenesis, a PPI network centered around genetic risk factors was generated. A list of genes associated to AMD was obtained from literature analyses and the OpenTargets website (including an evidence-based scoring system to quantify risk, see Methods and Supplementary Data 2). As the intent was to generate a system-wide view of the disease, we included loci with a relatively low score, since they could be part of a pathway that otherwise will be lost, and omission of pathways would lead to more of a distortion of the overall picture than the inclusion of a potentially noncritical pathway. Using gene-expression data, 13 genes (CTRB1, C20orf85, CTRB2, MTRNR2L13, SLC44A4, FRK, MBL2, TRPC4, OTOS, OR7G3, F13B, ASPM, and APOH) that were not expressed in neural retina and/or RPE-choroid^30^ were filtered out (Supplementary Data 3). As observed before,^31^ AMD-risk genes tend to be expressed in most of 37 non-retinal body tissues (see Methods); no AMD-risk gene is exclusively expressed in the retina (Supplementary Fig. 1a). If expressed in neural retina and/or RPE-choroid, AMD-risk genes tend to be more highly expressed in the RPE-choroid than in the neural retina (Supplementary Fig. 1b).

To gain insights into the networks that are possibly perturbed in AMD, direct binary protein interactions for all 130 AMD-risk gene products (proteins) expressed in neural retina and/or RPE-choroid were retrieved from the protein interaction databases HIPPIE, HuRi, and INTERACTOME 3D (Supplementary Data 4; see Methods). INTERACTOME 3D was additionally used to indicate binary interactions supported by 3D structural evidence, either an original protein structure, or by a homologous structure, or by a model inferred based on predicted domain–domain interactions. As ARMS2 is a risk protein, but no binary interactions were available in public databases, we added binary interactions from two-hybrid experiments from a recent publication.^32^ For 19 risk proteins (ADAMTS9, TSPAN10, TRPM3, CNN2, C6orf223, NT5DC1, SCPEP1, UHRF1BP1L, TYR, TDRP, FSTL5, EYS, FGD6, GALNT17, PDE1C, ZNF700, ABCA7, HMCN1, and PON1) no binary protein interactions were identified and they were deleted from the network. The remaining 112 AMD-risk protein connect to 1643 binary interactions, out of which 114 interactions are associated with 3D structural evidence. This network was further filtered by removing interactors not expressed in neural retina and/or RPE-choroid. This caused two risk proteins to be removed (RREB1 and FADS3). In total, the final binary network consisted of 110 AMD-risk proteins with 1426 interactors.

**Fig. 3:**
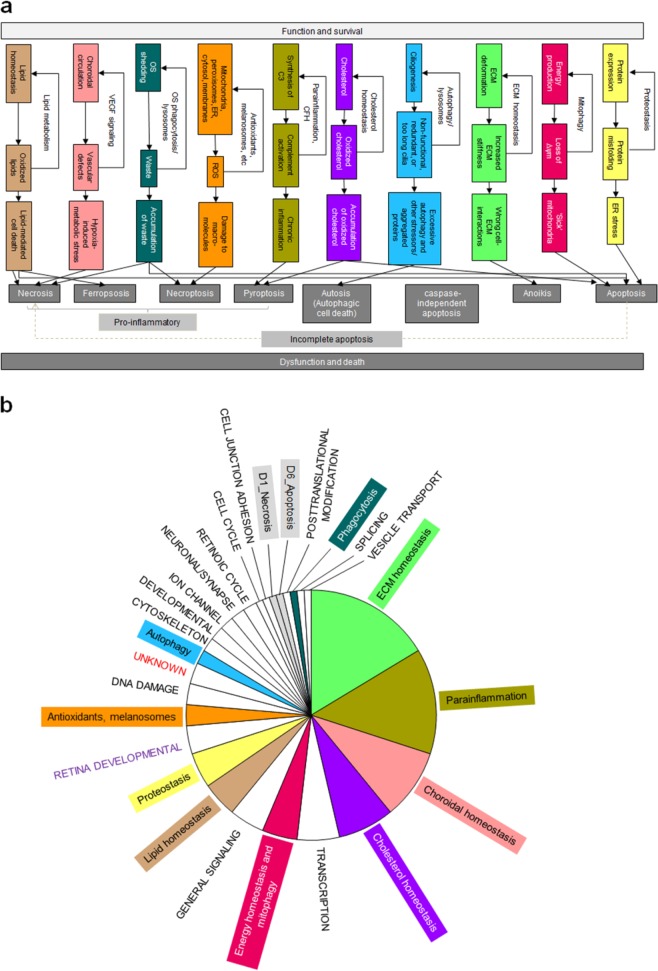
Processes involved in cell homeostasis and cell death. **a** Proteostasis describes the regulation of protein levels and their regulated degradation and is of primary importance in cells. Energy homeostasis and mitophagy are critical processes as the outer retina is the most energy-/ATP-requiring tissue in the body. ECM homeostasis describes the finely tuned processes of production and degradation of ECM components that are essential to ensure the mechanical homeostasis of tissues. Dysfunctional and failed cell–ECM contacts can cause cell death through anoikis. Autophagy is a critical cell homeostatic process that removes nonfunctional, redundant cilia that are too long, as well as aggregated proteins or organelles via the lysosomal machinery. However, autophagy may also cause cell death (autosis) under certain conditions. Cholesterol homeostasis is a critical process as the amount of membrane needed for the genesis of the outer disks is immense. Parainflammation describes the basal immune activation that the retina has because of its immune privilege, where under normal conditions microglia, perivascular macrophages, dendritic cells, and the complement system are basally activated in response to day to day damage and contribute to retinal homeostasis. Antioxidants are critical as ROS are generated from both internal (e.g., resulting from mitochondrial metabolism, outer segment phagocytosis, and NADPH oxidases) and external sources (e.g., light, smoking and high glucose) sources. Outer segment phagocytosis (heterophagy) and degradation of intracellular components (autophagy) requires lysosomal-mediated waste clearance. Choroidal vascular homeostasis and choroidal circulation are indispensable for mass exchange (nutrients, oxygen, etc.) with the outer retina. Hypoxia and associated metabolic stress in RPE cells can cause PR cell death. Dysfunctional lipid homeostasis can cause apoptosis, necroptosis, or ferroptosis. **b** Functional classes for the 110 AMD-risk proteins in the quantitative binary AMD network. The function for each risk protein was manually analyzed be detailed literature analyses and, if possible it was assigned to one of the 10 homeostasis functions or to one of the 7 modes of cell death. If this was not possible, it was assigned to other functional categories, such as transcription, general signaling, cytoskeleton, retinoic cycle, etc.

It is likely that progressive failure of homeostasis functions with age is an important component of AMD pathogenesis.^24^ Homeostatic processes (HP) operate to minimize the impact of perturbations affecting retinal cells, RPE, Bruch’s membrane, and choroid. Failure of homeostasis arising from aging and disease can lead to functional impairment and cell death. To link our binary protein interaction network to those homeostasis and cell death functions, we used ten previously defined functional homeostasis classes together with their link(s) to different modes of cell death (Fig. 3a; Supplementary Data 4).^24^ Those are proteostasis (HP1), energy homeostasis and mitophagy (HP2), ECM homeostasis (HP3), autophagy (HP4), cholesterol homeostasis (HP5), parainflammation (HP6), antioxidants (HP7), outer segment phagocytosis (heterophagy) and degradation of intracellular components (autophagy) and lysosomal-mediated waste clearance (HP8), choroidal vascular homeostasis (maintenance of adequate tissue perfusion without pathological angiogenesis—HP9), and lipid homeostasis (HP10). Using databases and manual literature searches we assigned each risk gene in the network, whenever possible, to one of the ten homeostasis functions or to one of seven modes of cell death [(Necrosis (D1), Necroptosis (D2), Pyroptosis (D3), Autosis (D4), Anoikis (D5), Apoptosis (D6), and Ferroptosis (D7)]. The homeostasis functions more tightly linked to AMD pathogenesis are ECM homeostasis, parainflammation and choroidal vascular homeostasis (Fig. 3b). Manual literature searches and the UniProt database (https://www.uniprot.org/) linked function to the 1426 interactors in the binary quantitative AMD network. Interestingly, one-third of the interactors (518 proteins) in the network can be assigned to one of the ten homeostasis or to the seven modes of cell death (Supplementary Fig. 2). The remaining two-thirds of the risk protein interactors were assigned to other cellular functions (Supplementary Fig. 2).

The most frequently identified functional classes of genes are proteostasis, ECM homeostasis, apoptosis, parainflammation, choroidal vascular homeostasis, and energy homeostasis. We represented the 110 AMD-risk genes that are in the final quantitative binary AMD network with increasing size of nodes following increasing confidence of genetic association to AMD (Supplementary Fig. 3). Eight of the ten homeostasis classes have at least one of the genes with the highest association to AMD. The classes of ECM homeostasis, cholesterol homeostasis, and parainflammation contain more than one high confidence risk gene. It is noteworthy that choroidal vascular homeostasis and energy homeostasis show high-risk associations with AMD.

### Direct binary PPI network representation of AMD and subnetworks

**Fig. 4:**
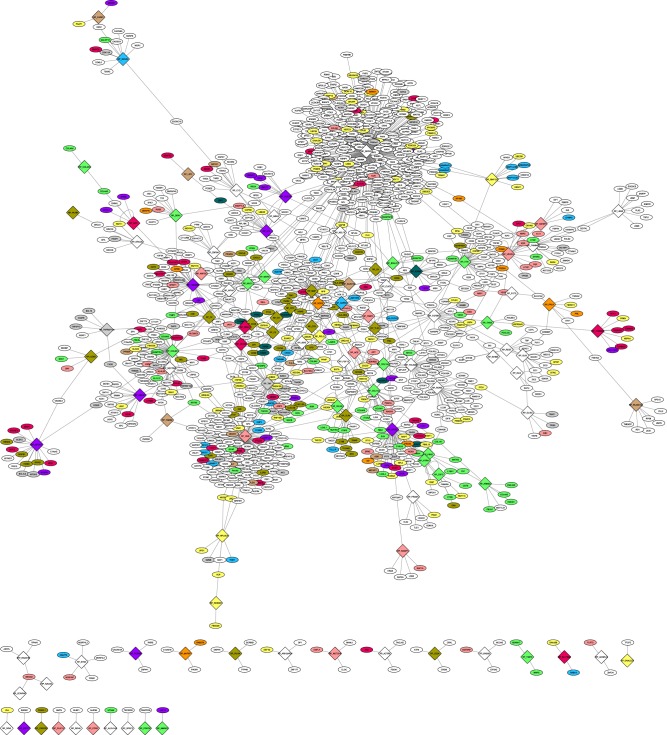
Overall topology of the binary quantitative AMD network. AMD-risk proteins are represented as diamonds and interacting proteins that are not AMD-risk proteins are shown as ellipse. Nodes are colored according to the ten homeostasis functions using the same color code as before. Cell death functions are indicated in gray and all other functions in white. For additional details see Data Availability Supplementary File 4 (can be viewed using Cytoscape software).

Cytoscape version 3.6.0 was used to represent the quantitative binary AMD network (Fig. 4; see Data Availability, Supplementary File 4). The network adopts a typical “scale free network” configuration with a high number of nodes that have few interaction partners and a few nodes with a very high number of interactors (SRPK2: 378; HGS: 123; MBP: 48; APOE: 45; COL8A1: 45). Functional homeostasis classes are highly intermixed. Nevertheless, two distinct clusters appeared for parainflammation and for ECM homeostasis, suggesting that these two disease modules may highlight different underlying AMD pathologies. The classes of choroidal vascular homeostasis and energy homeostasis are distributed throughout the network, suggesting that they do not act as individual disease modules, but together with other homeostasis functions. This important observation aligns with an emerging view that bioenergetics is an important determinant of homeostasis and repair mechanisms in general. In other words, it costs to continually repair the accumulated damage and loss of homeostasis seen in aging.

Focusing on the two homeostasis processes ECM and parainflammation (that included the two highest risk genes ARMS2/HTRA1 and CFH, respectively) resulted in a network of 346 proteins and 360 interactions. While interactors are enriched in ECM homeostasis and parainflammation functions, all other homeostasis functions appear in the network (and in total 48 AMD-risk proteins are present in this subnetwork) (Supplementary Fig. 4). This suggests that a functional impairment of ARMS2/HTRA1 or CFH not only disturbs ECM and complement functions, but ultimately is likely to affect all cell and tissue homeostasis functions. Central node risk genes are often expressed with lower abundance than the average of their interaction partners. Thus, increasing or decreasing protein levels through polymorphisms may have significant impact on the networks in proximity of the perturbed node. Importantly, there are many links to choroidal vascular homeostasis, with a higher fraction linking to EMC homeostasis compared to parainflammation (16 vs. 8 interactions). Interestingly, the ARMS2/HTRA1 locus was significantly more associated to neovascular disease as compared to geographic atrophy.^7^

### Integrative analysis of the SBGN model and the PPI network

To compare proteins included in the SBGN model with proteins included in the PPI network, we broke down all entities in the SBGN model that were included as complexes into proteins and combined them with the remaining protein and RNA entities in the SBGN model. This resulted in a total set of 952 proteins (=“SBGN model proteins”). Comparing this set with the 1314 proteins in the PPI network (=“PPI network proteins”), resulted in an overlap of 205 proteins that are included in both, the SBGN model and the PPI network (Supplementary Fig. 5). We also generated a list of 2060 proteins that are either in the SBGN model or in the PPI network or in both (=“SBGN-PPI union proteins”) (Supplementary Data 5).

**Fig. 5:**
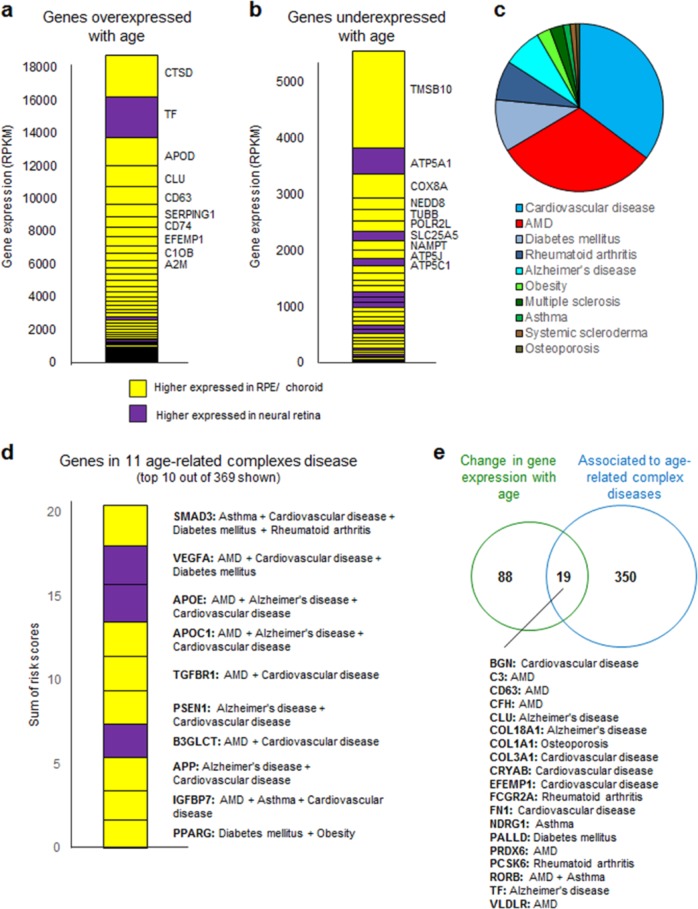
Analyses of the 2060 SBGN-PPI union proteins. **a** Genes overexpressed with age and their respective expression levels in neural retina (purple) or RPE-choroid (yellow) are shown. **a** Genes underexpressed with age and their respective expression levels in neural retina (purple) or RPE-choroid (yellow) are shown. **c** Pie chart of sums of risk scores for all 2060 SBGN-PPI union genes in eleven age-related complex diseases. **d** Top ten genes with highest risk score in eleven age-related complex diseases. **e** Overlap of genes changing with age and associated to at least one age-related complex disease.

Gene expression is known to be altered in ageing organisms. As AMD is linked to ageing, we analyzed a meta-analysis of age-related gene-expression profiles.^33^ We found that the 2060 SBGN-PPI union proteins are significantly enriched in proteins where the corresponding genes have been found to be either up or downregulated with age (108 of 2060 compared to 347 genes changing with age in the whole genome; *p* value < 1e−5, Fisher’s exact test). Combining this set with gene-expression analyses in neural retina and RPE/choroid,^30^ enabled us to generate a list of genes that are high expressed and change with age (Fig. 5a, b). The top ten overexpressed genes are CTSD, TF, APOD, CLU, CD63, SERPING1, CD74, CRYAB, EFEMP1, C1QB, and A2M. The top ten underexpressed genes are TMSB10, ATP5A1, COX8A, NEDD8, TUBB, POLR2L, SLC25A5, NAMPT, ATP5J, and ATP5C1. Interestingly those genes are mostly RPE-choroid specific genes, suggesting that age-related changes mainly manifest in the RPE/choroid and less in the neural retina.

Next, we retrieved genes associated with increased risk of developing ten other age-related complex diseases from the OpenTargets database (Alzheimer’s disease, Multiple sclerosis, Osteoporosis, Systemic scleroderma, Asthma, Cardiovascular disease, Diabetes mellitus, Obesity, and Rheumatoid arthritis) (Supplementary Data 5) and combined these with the AMD-risk genes. We found that the 2060 SBGN-PPI union proteins are enriched in proteins, where the corresponding genes have been found to be associated to eleven complex diseases (369 of 2060 compared to 2134 genes associated to age-related complex disease in the whole genome; *p* value < 1e−5, Fisher’s exact test). Interestingly, the summed risk score for all genes among the 2060 that are associated to cardiovascular diseases is even higher than the summed score for AMD-risk genes, suggesting that individuals with cardiovascular diseases might be at a higher risk for AMD and vice versa (Fig. 5c). Shared pathogenic mechanisms between AMD and cardiovascular disease were proposed earlier.^34^

Other complex diseases that have high-risk scores are diabetes mellitus,^35^ rheumatoid arthritis, and Alzheimer’s disease.^36^ The top ten genes that have the highest risk score sum of all eleven age-related complex diseases are PPARG, IGFBP7, TGFBR1, PSEN1, B3GLCT, APP, APOC1, APOE, VEGFA, and SMAD3 (Fig. 5d). In total, 19 proteins are associated with genes that change their gene-expression levels with age and that are associated with any of the 11 complex diseases (Fig. 5e).

## Discussion

Systems biology provides tools to integrate diverse data types including molecular and pathway information to predict disease outcomes and is becoming an increasingly powerful and popular tool in drug discovery and development.^37^ Modeling can be used to reveal system behaviors that are otherwise difficult to isolate or are expensive or burdensome on resources to conduct experimentally. In the present study, we describe two network-centric approaches to capture the potential molecular mechanisms of AMD pathogenesis. The AMD SBGN model is a spatially ordered map of known AMD pathogenesis and as such is a biological knowledge driven model. In contrast, the AMD PPI model is centered on the AMD-risk gene products and thus represents a systems approach. SBGN has evolved as a method of mapping whole networks comprehensively, representing interconnections and making it easy to visually identify overlaps in pathways within the network. Software such as CellDesigner has been developed allowing for the creation of large comprehensive maps of signaling pathways and metabolic processes, which can be used to explore how the different elements and pathways in the network may influence one another—an otherwise difficult and nonintuitive task, that could provide insight into new areas for investigation. While the main aim of our work was to collate knowledge on AMD pathology in a graphical way, the ability to integrate simulation software into CellDesigner (or use the SBGN models in other simulation software, e.g., COPASI^38^) provides the possibility to perform more detailed quantitative computational analysis of aspects of AMD pathogenesis in the future.

The AMD PPI network links 110 AMD-risk proteins expressed in neural retina and/or RPE-choroid to 1426 proteins via direct binary protein interactions that were retrieved from databases and literature searches. The PPI network contains links to homeostasis functions, such as proteostasis, energy homeostasis/mitophagy, ECM homeostasis, autophagy, cholesterol homeostasis, antioxidants, phagocytosis, choroidal vascular homeostasis, and lipid homeostasis. Our PPI network shows that although the functional homeostasis classes are highly intermixed, two distinct clusters appeared for parainflammation and for ECM homeostasis, suggesting that these are two disease modules which may represent different underlying molecular pathologies in different AMD patients. Interestingly, the choroidal vascular and energy homeostasis functions are distributed throughout the network, suggesting that they do not act as individual disease modules, but work together with other homeostasis functions. This important observation aligns with an emerging view that bioenergetics is an important determinant of homeostasis and repair mechanisms in general.

Our combined analysis of the two types of models shows that the 2060 “SBGN-PPI-union” proteins coding genes significantly overlap with genes that change their expression with age. Interestingly, a large fraction of those proteins also turned out to be associated with other complex diseases, such as cardiovascular disease, Alzheimer’s disease, rheumatoid arthritis, or asthma. Genetic pleiotropy between AMD and other complex diseases has been observed before.^39^ The emerging question is, if there is such a commonality for these diseases in terms of their genetic makeup, what determines why one individual develops one and not all diseases? One reason may be that there are different polymorphisms in a single gene that create different risks for different diseases. However, similar variants in the same gene could also be protective for one disease and confer risk for another disease, such as demosntrated for APOE in AMD and Alzheimer’s disease.^40^

There are certainly limitations about the network-centric approach that we have taken in this work. First, the PPI networks that are currently stored in databases are largely derived from experiments that aimed to study intracellular networks and that often were performed in transformed cell lines. Thus, we only have sparse information on networks across cells and tissues. Second, the PPI datasets stored in database were mostly generated using immortalized cell lines, overexpressed proteins, or two-hybrid approaches, which can force proteins to interact that normally would not encounter each other in a cell, or would interact only under specific conditions or in certain cell types. Therefore, some reported interactions may not be physiologically relevant for the retinal systems under consideration here. Third, we did not account for positive (protective variant) or negative (risk variant) effects of the genes, as it is not possible to predict what is the impact of a variant on protein function as the majority of them are located in noncoding intron regions of the genes. A protective risk variant could cause an increase in protein level/activity for one gene and a decrease in protein level/activity for another gene. Finally, the complexity of AMD lead to the inevitable challenge that no attempt to represent the complexity in SBGN or indeed any other format can be totally comprehensive. We hope that making the current version available will allow investigators to extend at will.

Our work highlights the need for transformational approaches to understand and advance the understanding of complex diseases.^41^ Importantly, the approach we have taken here already generates knowledge that is beyond what one puts in (in contrast to standard modeling approach that is purely knowledge driven). Our two representations of AMD may be incorporated into quantitative whole-cell energy balance models that aim to calculate what it costs to continually repair the accumulated damage and predict how during different homeostasis functions are balanced and how repair failure may cause different modes of cell death. In this way, modeling can help to broaden our knowledge, generate new hypotheses and pinpoint interesting and/or new areas for analysis. This may in future be used to guide in vitro experiments that aim to improve cellular and tissue repair mechanisms that may delay age-related accumulation of damage.

## Methods

### Identification of AMD-risk genes

To assemble a list of genes that are associated to the risk of developing AMD, we analyzed genes discovered in 13 genome-wide association studies (GWAS) with, usually, large samples sizes (Supplementary Data 2). In addition, we analyzed 37 publications addressing family linkage, exome sequencing, and candidate gene testing studies of AMD cases vs. controls (Supplementary Table 2). This resulted in a total of 144 AMD-risk genes (11 genes known to cause monogenic early macular degenerations were excluded). We adapted a quantitative scoring system based on the OpenTargets initiative of the European Bioinformatics Institute (EMBL-EBI) (https://www.targetvalidation.org), where the score of the risk genes depends on factors that affect the relative strength of available evidence, for example *p* values and sample size for the GWAS data (Supplementary Fig. 6). We chose to adopt this broader-based approach over simply taking the results of the latest major GWAS study,^7^ in order to optimize chances of capturing potentially relevant pathways.

### AMD pathogenesis and its graphical representation

The literature was searched for studies addressing the pathogenesis of AMD. A comprehensive systematic review of evidence was not considered feasible but by taking a more targeted approach and focussing on reviews we sought to achieve a comprehensive overview of the current state of knowledge. Systems Biology Graphical Notation^21^ was considered to be the most appropriate vehicle for representing the complexity of AMD pathogenesis. CellDesigner (http://www.celldesigner.org/) offers a flexible descriptive, XML-coded structured editor with multiple classes of molecular species and a variety of reaction types, which are represented by differing arrow types, and relate to biochemical and gene regulatory networks and transport of species between compartments.^38^ It also supports the computational simulation of networks and parameter estimation by an integration with SBML ODE Solver, SBML Simulation Core, and Copasi.

To ensure that we achieved the maximum possible descriptive power, we used the enhanced feature set within CellDesigner that represents a superset of the strict SBGN syntax. We also used the “phenotype” category flexibly, to relate to sets of pathways represented elsewhere within the model as well as more classical phenotypic descriptors. File references are given in red, an internal pathway in dark green and actual phenotypes in the default purple. CellDesigner allows for choosing and arranging of compartments in a similar way to that of the true anatomy. We elected to use a relatively fine-grained anatomical compartmentalization recognizing that although precise localization would not be possible for all molecular species at all sites there are some elements of AMD pathogenesis that are highly spatially restricted and in a way that may be critical to understanding the disease. We did not, however, explicitly distinguish macula from more peripheral retina.

One of the key utilities of SBGN and other modeling frameworks is the potential for reuse of published models and we searched for existing models that might inform our AMD representation. We identified the SBGN model of Parkinson’s disease^42^ and an ODE model of complement activation^43^ as sources of data. In both instances the pre-existing models were modified to take into account patterns of gene expression and, in the case of the complement model, we replaced the pathogen component of the model with altered host reflecting the likely mode of complement activation in the sub-RPE—choroid space.

### Gene expression of 144 AMD-risk genes in ocular tissues

Despite advances in mass spectrometry, the comprehensive identification of all biologically significant proteins and isoforms in a complex mixture (e.g., human primary cells or tissues) has not been achieved to date. Therefore, we used mRNA expression as a proxy for protein levels and in particular we exploited the following published datasets: (i) the Whitmore et al., 2014 dataset,^30^ which contains RNA expression levels (by RNA sequencing) from RPE-choroid and neural retina of four human donor eyes; and (ii) gene-expression levels (by RNA sequencing) in total retina (Fantom 5 database, http://fantom.gsc.riken.jp/5/). We took the top 95% most highly expressed genes in each respective dataset to identify those considered significantly expressed in the RPE-choroid or neural retina.

### Gene expression of 144 AMD-risk genes in non-retinal tissues

Expression level of 37 non-retinal tissues (HPD database; https://www.proteinatlas.org) were analyzed by classifying the AMD-risk genes, according to the number of non-retinal tissues each gene is expressed in, into four groups: (i) T1, expressed in ≥30 of 37 non-retinal tissues, (ii) T2, expressed in ≥15 <30 non-retinal tissues, (iii) T3, expressed in ≥1 <15 non-retinal tissues, and (iv) not expressed in any non-retinal tissue.

### Protein interaction databases

The following public databases of protein–protein interactions were interrogated: HIPPIE [http://cbdm.uni-mainz.de/hippie/], HuRi [http://interactome.baderlab.org/search] and INTERACTOME 3D [https://interactome3d.irbbarcelona.org/]. Direct binary interactions (no complexes) were ascertained. The networks were represented in Cytoscape and the topological properties were analyzed using the Cytoscape “Network Analysis”.

## Supplementary information

Supplementary Information

Supplementary Data 1

Supplementary Data 2

Supplementary Data 3

Supplementary Data 4

Supplementary Data 5

## Data Availability

All data are provided in the manuscript, Supplementary information, and in a UCL research data repository. The UCL research data repository can be accessed at https://www.ucl.ac.uk/isd/services/research-it/research-data-repository; 10.5522/04/10303694. AMD maps are listed as Supplementary Files 1–4 in the UCL research data repository.
