# Supplementary material for: Repository of proposed pathways and protein–protein interaction networks in age-related macular degeneration
Source: NPJ Aging Mech Dis. 2020 Jan 7;6:2. doi: 10.1038/s41514-019-0039-5 (PMC6946811; doi:10.1038/s41514-019-0039-5)
Supplement: Supplementary file 1 — Supplementary Information [file 41514_2019_39_MOESM1_ESM.pdf]

Supplementary Information

Repository of proposed pathways and protein-protein interaction networks in age-related macular degeneration

Authors: Fran M. Pool, Christina Kiel, Luis Serrano, Philip J. Luthert

Content:

Supplementary Figures

Supplementary Figure 1. Gene expression of 144 AMD risk genes in retinal (neural retina and/or RPE/choroid) and non-retinal tissues.
Supplementary Figure 2. Functional classes for the 1426 interactors of the AMD risk proteins of the quantitative binary AMD network.
Supplementary Figure 3. Function of 110 AMD risk protein of the binary network and genetic risks.
Supplementary Figure 4. The ECM homeostasis and parainflammation sub-network.
Supplementary Figure 5. Overlap of proteins in SBGN model and PPI network.
Supplementary Figure 6. Distribution of quantitative scores for 144 AMD risk genes.

Supplementary Tables (provided as additional excel data files)

Supplementary Data 1. List of publications used to inform the SBGN model.
Supplementary Data 2. List of genes linked to AMD.
Supplementary Data 3. Gene expression for 144 AMD risk genes in ocular tissues (neural retina and RPE/choroid)
Supplementary Data 4. Direct binary protein interactions for all 130 AMD risk gene products (proteins) expressed in neural retina and/or RPE/choroid.
Supplementary Data 5. Integrative analysis of the SBGN model and the PPI network.

Supplementary Files (provided as additional files)

Supplementary File 1. AMD SBGN model (xml file)
Supplementary File 2. Mitochondria sub-module SBGN model (xml file)
Supplementary File 3. Macrophage sub-module SBGN model (xml file)
Supplementary File 4. PPI network cytoscape file (cys file)

a

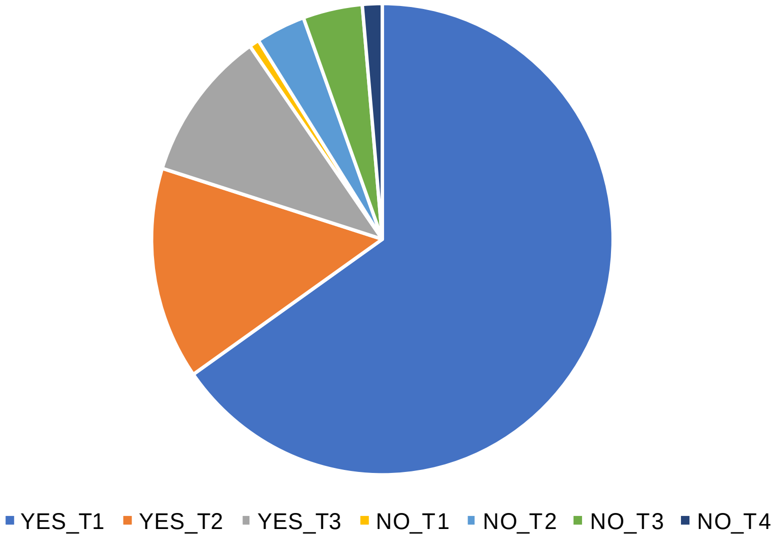

b

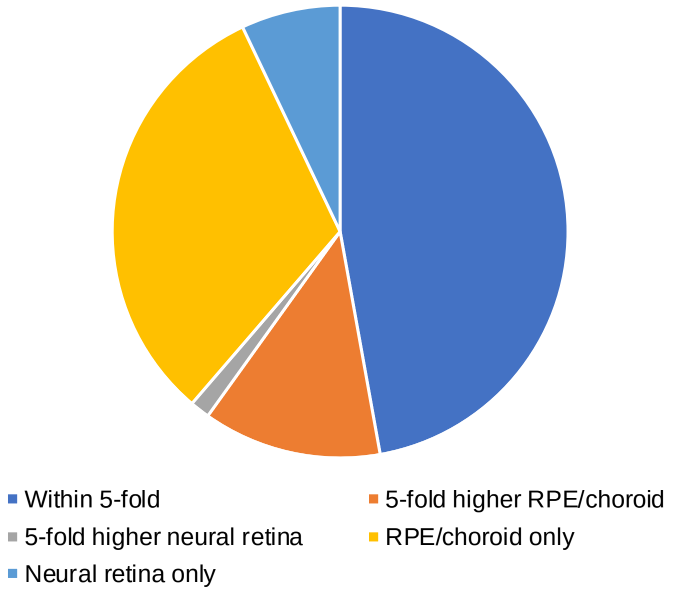

Supplementary Figure 1. Gene expression of 144 AMD risk genes in retinal (neural retina and/or RPE/choroid) and non-retinal tissues. (**a**) Quantitative gene expression for 144 AMD risk genes in RETINA and 37 non-retinal tissues. For the classification of the 144 AMD risk genes the expression information in retina (YES/ NO) was combined with the expression information in 37 non-retinal tissues (T1: expressed in >=30 of 37 non-retinal tissues; T2: expressed in >=15 <30 non-retinal tissues; T3: expressed in >=1 <15 non-retinal tissues; T4: not expressed in any non-retinal tissue). (**b**) REPR/choroid vs neural retina expression of AMD risk genes. Based on the Whitmore et al 2014 dataset AMD risk genes were classified based on their quantitative expression levels in RPE/choroid and neural retina. The source data associated to this figure are provided in Supplementary Data 3.

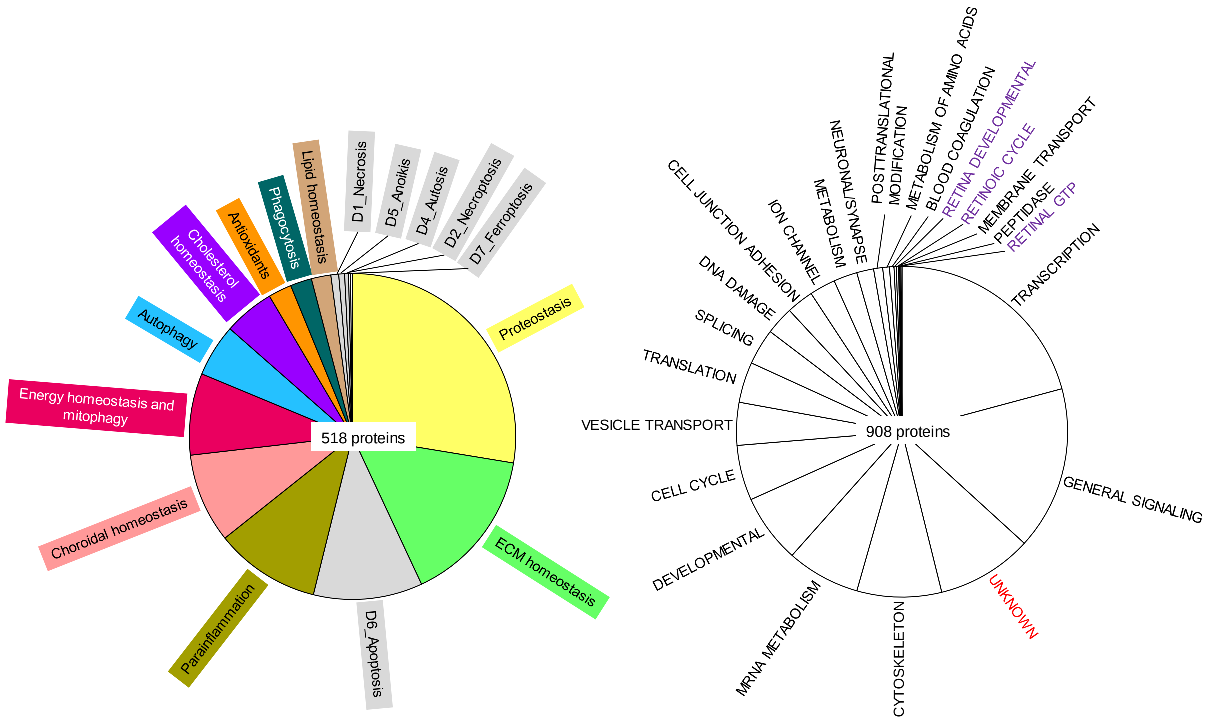

Supplementary Figure 2. Functional classes for the 1426 interactors of the AMD risk proteins of the quantitative binary AMD network. The function for each interactor protein was manually analysed be detailed literature analyses and, if possible it was assigned to one of the 10 homeostasis functions or to one of the 7 modes of cell death (left side). If this was not possible, the interactor protein was assigned to other functional categories, such as TRANSCRIPTION, GENERAL SIGNALING, CYTOSKELETON, MRNA METABOLISM, DEVELOPMENTAL, CELL CYCLE, VESICLE TRANSPORT, etc (right side). The source data associated to this figure are provided in Supplementary Data 4.

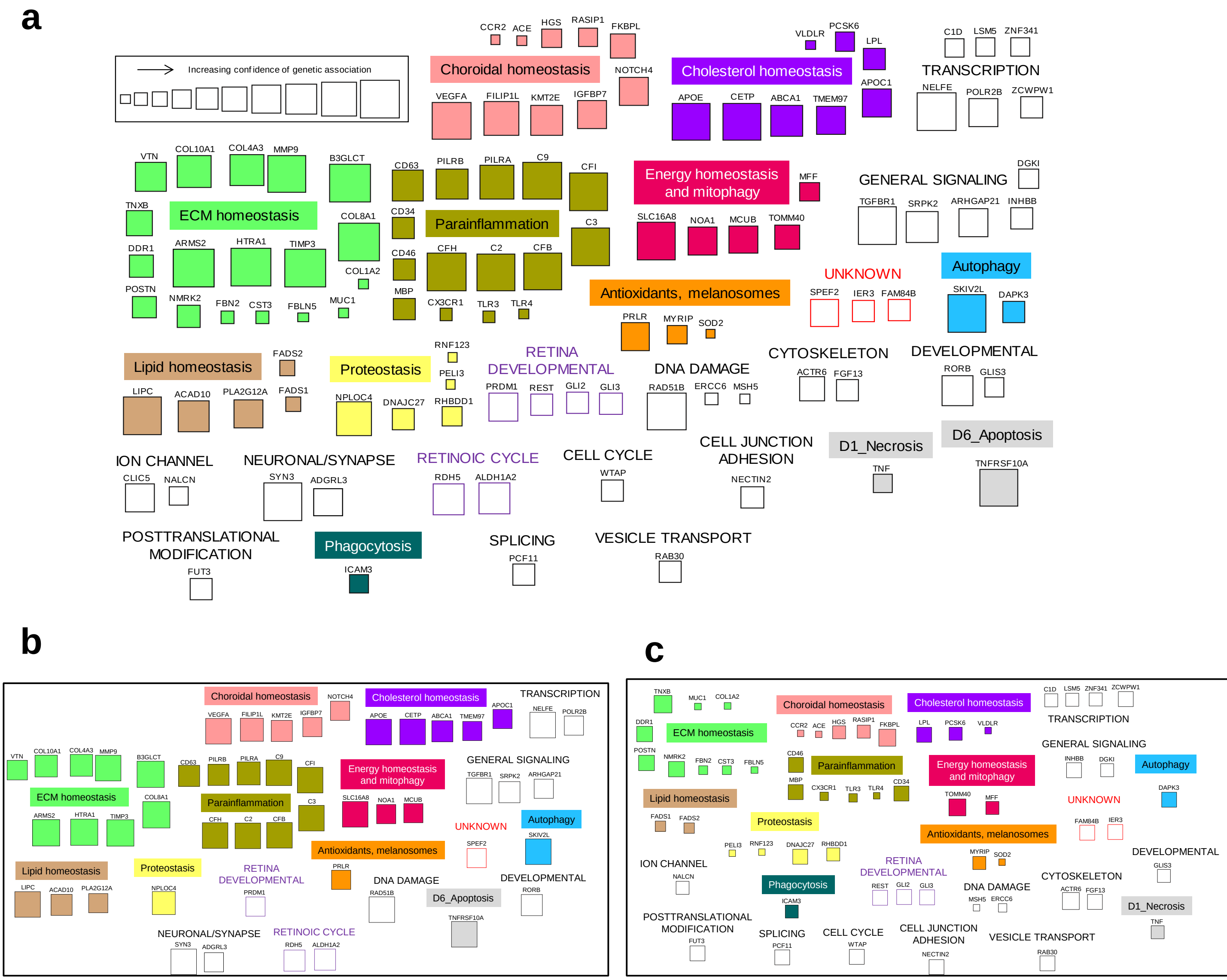

Supplementary Figure 3. Function of 110 AMD risk protein of the binary network and genetic risks. (**a**) The different colours correspond to different homeostasis functions (see also Figure 3). The size of the squares corresponds to the confidence of genetic association to AMD. (**b**) Similar as in (a), but only high risk genes (>=0.7 risk score are shown). (**c**) Similar as in (a), but only risk genes with a score of < 0.7 are shown. The comparison of the three panels shows that the functional classes/ pathways contain both, genes with high and low risk variant scores. Thus, the types of pathways implicated are relatively robust with respect to the genetic risk score, suggesting that only a certain number of pathways are affected and that some risk variants that have a lower score are also part of a specific disease pathway/network module.

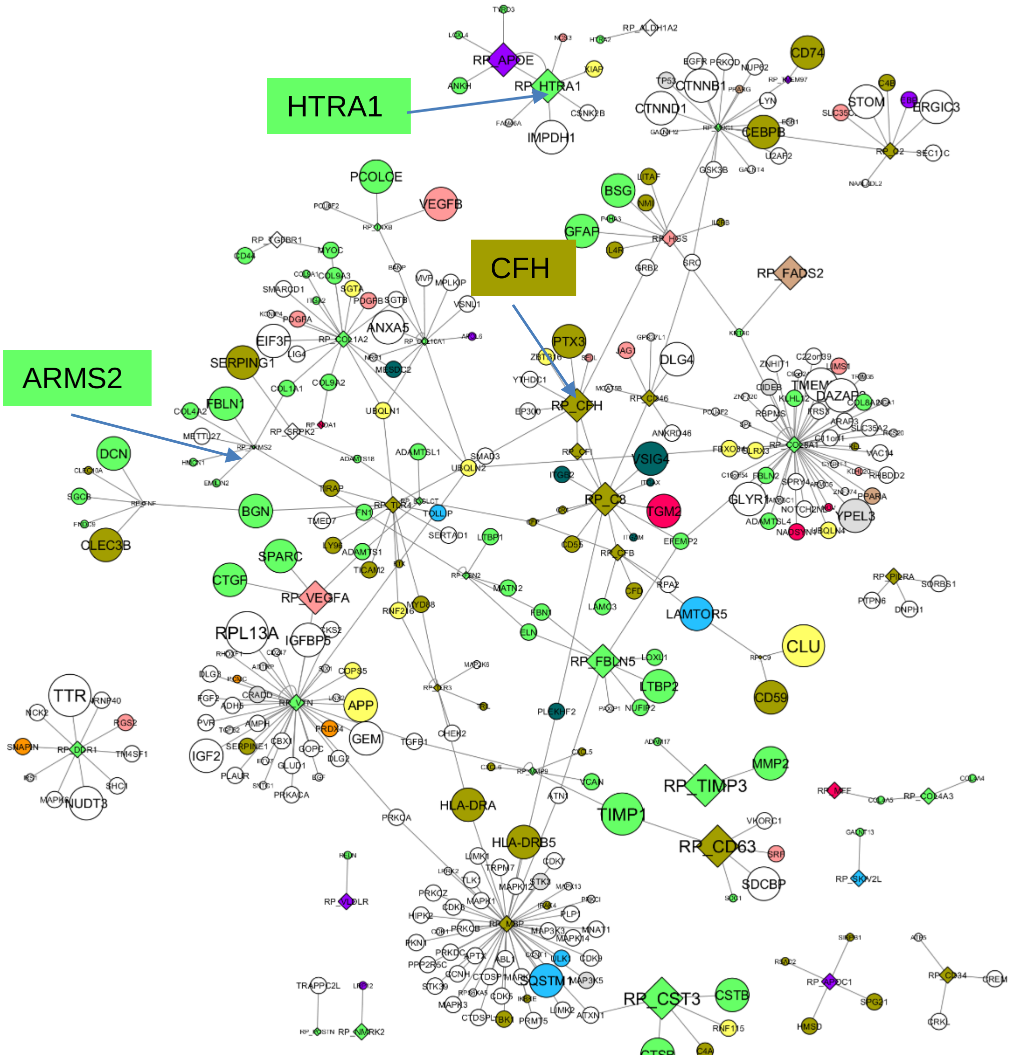

Supplementary Figure 4. The ECM homeostasis and parainflammation sub-network. AMD risk proteins are represented as diamonds and interacting proteins that are not AMD risk proteins are shown as ellipse. Nodes are coloured according to the 10 homeostasis functions using the same colour code as before. Cell death functions are indicated in grey and all other functions in white. The node size corresponds to the maximum expression in retinal tissue (RPE/choroid or neural retina). The source data associated to this figure are provided in Supplementary Data 4 and in Supplementary File 4 as a cytoscape file that can be read using the Open-Source programme Cytoscape (https://cytoscape.org/).

.

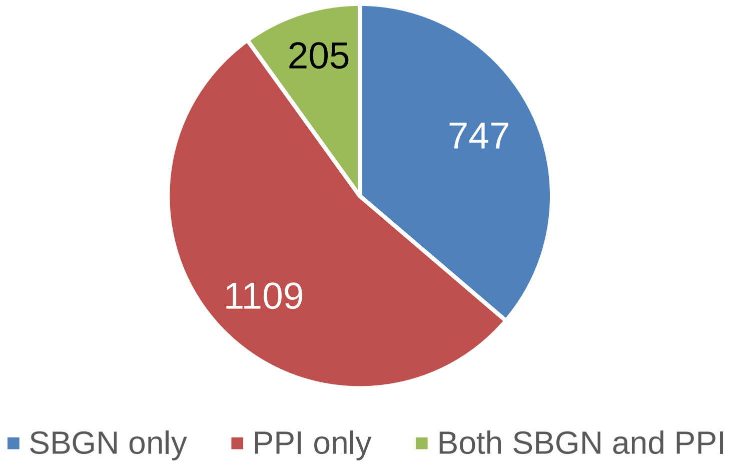

Supplementary Figure 5. Overlap of proteins in SBGN model and PPI network. Pie chart diagram of overlap of proteins the two types of networks representations. The union of proteins that are either in the SBGN model or in the PPI network or in both (=’SBGN-PPI union proteins’) is 2060. The source data associated to this figure are provided in Supplementary Data 5.

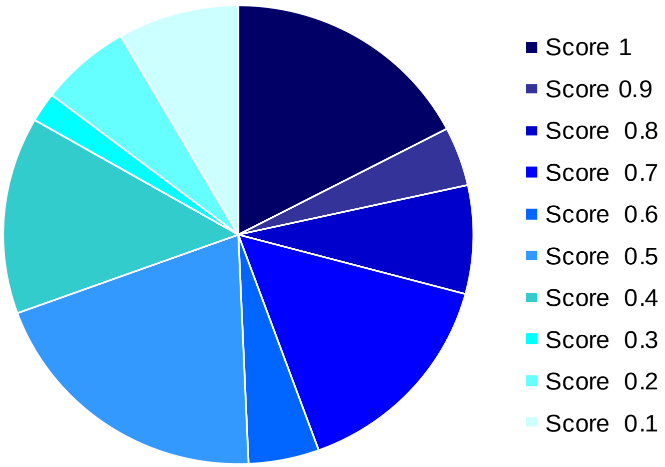

Supplementary Figure 6. Distribution of quantitative scores for 144 AMD risk genes. A higher score reflects a higher likelihood of the risk gene to contribute to the AMD phenotype. The source data associated to this figure are provided in Supplementary Data 2.
